# Pilot study on the functional relevance of single versus double toe transfer in monodactyly: Analysis on functional integration and grasping

**DOI:** 10.1016/j.jpra.2025.12.021

**Published:** 2025-12-24

**Authors:** Veronica Fasoli, Chiara Parolo, Elisa Rosanda, Rossella Pagliaro, Silvia Minoia, Francesca Tolosa, Luigi Troisi, Giorgio Eugenio Pajardi

**Affiliations:** University Department of Hand Surgery and Rehabilitation, San Giuseppe Hospital, IRCCS MultiMedica Group, Via S. Vittore 12, 20123, Milano, Italy

**Keywords:** Toe transfer, Double toe transfer, Symbrachydactyly, Congenital hand, Free phalangeal transfer

## Abstract

Monodactyly is a rare congenital anomaly of the upper limb, characterized by the presence of an isolated thumb. Although toe-to-hand transfer is a well-established surgical reconstructive procedure, there are currently no universally accepted guidelines clearly defining the optimal number of digits to be transferred. The aim of this pilot study is to evaluate whether performing a double toe transfer offers a significant functional advantage over a single toe transfer. We collected seven cases which were divided into two groups based on the number of digits transferred. All patients underwent the same standardized surgical and rehabilitative protocol. During follow-up, the motor patterns of each patient and the integration of the transferred digits during grasping were systematically evaluated.

In our series, while double toe transfer has enhanced grasp and function by providing a three-digit pinch, a single transferred toe combined with a structured thumb appeared able to perform most daily activities.

The second transferred digit has often shown to be stiffer as compared to the first toe transfer, limiting function while increasing donor site morbidity and operative time. Careful preoperative assessment of residual anatomy and spontaneous grasp strategies should guide surgical planning to maximize functional integration and minimize unnecessary morbidity. This study highlights the need for a selective, patient-specific approach in toe-to-hand transfer reconstruction in children affected by monodactyly.

## Introduction

Congenital anomalies of the upper limb can be classified according to the Swanson system introduced in 1964.^1^^,^^2^ This classification is based on disruption of embryological development and distinguishes between transverse and longitudinal defects, failure of differentiation of parts, duplication defects, anomalies due to overgrowth or undergrowth, congenital constriction band syndrome and generalized skeletal abnormalities.

Symbrachydactyly results from transverse arrest of limb development and shows a wide spectrum of clinical manifestations. In order to provide a more structured phenotypic framework, Blauth and Gekeler^3^ proposed a classification system, including short finger type, cleft hand type, monodactyly type and peromelic type.^4^

Monodactyly is characterized by the presence of an isolated thumb with absent or severely hypoplastic fingers. It may occur isolated or in the contest of a syndromic condition. The thumb may exhibit normal morphology and function, or it may be hypoplastic and/or unstable according to Foucher classification.^4^ The absence of opposing digits significantly compromises hand function. Surgical reconstruction aims to restore prehension and sensate pinch through toe-to-hand transfer.^5^^,^^6^

No uniform guidelines define the optimal number of digits to transfer in monodactyly. Some authors advocate the transfer of a single digit in ulnar position, aiming at creating a functional space between the thumb and the transferred digit to allow for effective grasping and pinch.^6^ Others have reported the transfer of two toes in an attempt to provide a three-digit pinch.^5^^,^^7^ When two toes are transferred, the digits can be positioned, either simultaneously or sequentially, into the middle and ring/small finger positions or, alternatively, into ring and small finger positions.^6^

However, clinical experiences reported in the literature suggest that transferred digits often exhibits a reduced active range of motion and consequently a poor grip function, particularly with regard to fine motor tasks.^8^ Furthermore, before surgery it remains uncertain how and if children will integrate both the transferred digits into their graps.^9^ The primary goal of this study was to investigate whether performing a double toe transfer offers a significant functional advantage over a single toe transfer in children affected by monodactyly. The secondary aim was to identify clinical and anatomical factors that could help guide surgical decision-making process.

## Materials and methods

A retrospective observational pilot study was conducted in April 2025 at the Hand Surgery Unit of San Giuseppe Hospital in Milan, aimed at analyzing the functional outcomes in children affected by monodactyly who underwent reconstruction of the missing digits through a single or a double toe transfer between January 2015 and April 2024. This manuscript followed STROBE guidelines.

A minimum of 1 year follow-up was required for inclusion. Children thumb agenesia were excluded from the study. To minimize bias, another exclusion criteria were patients presented with complex syndromic malformations as their condition could have influenced both motor development and functional integration of the transferred toes.

Preoperative radiographs were obtained to evaluate the presence and development of metacarpals and digitals bones. Serial radiographs were performed up to 2-3 years of age to monitor bone growth and guiding optimal placement of the transferred digit. Each child underwent at least one neuropsychomotor therapy session aimed at analyzing the strategic movement patterns adopted by the patient prior to surgical intervention. All patients received at least one psychological consultation to provide support to the family and to assess the underlying motivations and expectations regarding the surgical procedure. If necessary additional sessions were carried out to further support the family and refine the preoperative assessment.

The decision to perform a single or double toe transfer was based on preoperative clinical evaluation and radiographic imaging, with particular attention to residual digital anatomy and metacarpal development. In addition, in patients presenting a rudimentary second digit, free phalanx grafting from the foot was performed to enhance its function and support its role in grasping movements. In certain cases, although a double transfer was initially planned, intraoperative findings such as vascular anomalies of recipient vessels, led the surgeons to opt for a single transfer. The potential for a second transfer was left open but ultimately not pursued due to the satisfactory functional outcomes during follow-up.

If necessary, secondary procedures of tenolysis was performed after at least 1 year of surgery.

We performed a retrospective division in two groups A and B in order to allow for comparison between the functional outcomes of single versus double toe-transfer. All children were immobilized for the first postoperative week. Starting from the 8 day after surgery, a standardized rehabilitation protocol started, beginning with daily passive mobilization exercises. Over time, therapy progressed to include active mobilization and two neuropsychomotor therapy sessions per week until approximately the 8th postoperative week. At that time fixation devices were removed and, if indicated, the frequency of physiotherapy session was gradually reduced to three times per week. Functional assessments were derived from routine follow-up examinations performed both by the surgical team and physiotherapists, with a specific focus on grasp strategies and digit involvement. Since no validated pediatric scale specifically addressing digital integration after toe transfer is currently available, functional data were collected through direct clinical observation and systematically recorded. Any additional surgical revisions were documented.

## Results

Between January 2015 to April 2024, we collected seven cases of children affected by monodactyly who underwent digital reconstruction through toe-to-hand transfer. Among these, three children received a single stage procedure of double toe transfer to the hand (Group A), while four children underwent a single toe transfer (Group B). The mean follow-up was 6 years and 11 months, with a range between 1 year and 6 months and 10 years and 6 months.

Group A included one child with monodactyly and severe hypoplasia of the second digit, a boy with monodactyly and severe hypoplasia of the second and fifth digits, and the last one affected by hypoplastic thumb and agenesis of the remaining digits. This group consists of two males and one female. In all three patients, the affected hand was the left. The mean age at the time of surgery was 2 years and 4 months, with a range between 1 years and 11 months and 3 years and 2 months.

Group B included one girl with monodactyly and agenesis of other digits, and three children with monodactyly and a severe hypoplastic second digit. All four patients in this group also presented with the congenital deformity in the left hand. This group included three females and one male.

The mean age at the time of surgery was 3 years, with a range between 2 years and 8 months and 3 years and 5 months.

### Group A

**Figure 1:**
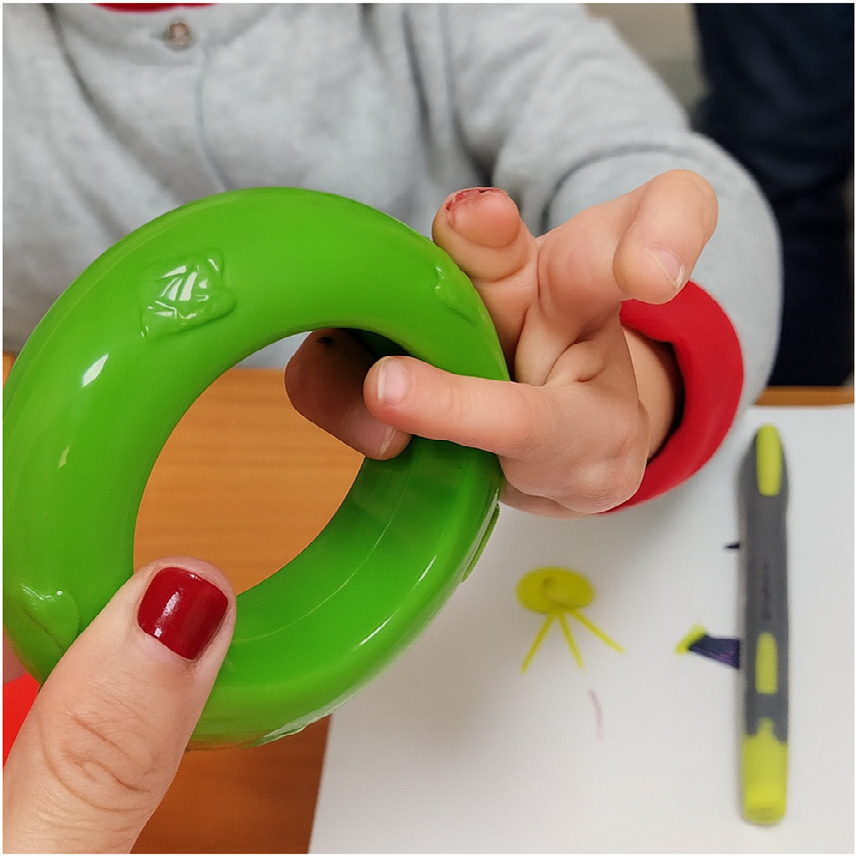
Preferential grasp patter in patient underwent double toe transfer and free phalangeal transfer. Due to stiffness of the centrally transferred digit, the patient adopts a functional grasp between the thumb and more ulnarly positioned transferred digit. The second hypoplastic digit is used as a stabilizer. The centrally placed transferred digit is completely excluded from the grasping pattern.

Since preoperative grasp analysis showed an attempt to use a thumb–hypoplastic finger pinch, the female patient with a rudimentary second digit underwent a free phalanx graft to enhance its function and simultaneously a double toe transfer. The toes were positioned on the fifth and the third metacarpal bones respectively. No further reconstructive procedures have been performed. However due to marked stiffness of the centrally positioned digit, teno-artrholysis surgery is currently being planned. During follow-up, the child consistently demonstrated a preference for a thumb-second digit pinch when manipulating small objects. Larger objects were grasped using a thumb-ulnar digit grip. The centrally transferred toe was functionally excluded from most manual tasks and only recruited as a stabilizer for large objects [Figure 1].

When she was asked to express a preference by ranking each finger in order of importance, she identified the rudimentary indicis as the most important digit, followed by the thumb, the transferred toe in ulnar position and lastly the centrally positioned transferred digit.

The one patient with rudimentary second and fifth digits underwent a double toe transfer and a free phalanx graft for the only fifth digit. Preoperative grasp observation showed that the second digit was poorly represented and rarely involved in grasping patterns, while the fifth digit had sufficient structure to be functionally reinforced through grafting.

The transferred toes were positioned on the third and fourth metacarpal bones, respectively. During follow-up the child predominantly used a thumb-digit grasp involving the toe transferred to the third metacarpal bone for handling small objects. The digit transferred to the fourth metacarpal bone was excluded from active grasping movements, serving mainly as a stabilizer. However, this last function was often compensated by the fifth digit. The patient underwent tenolysis of both transferred digits 1 year postoperatively. Eight years later, a second procedure of teno-arthrolysis was performed to remove the second rudimentary digit to widen the first web. Flexion of both digits after surgeries improved, but the overall grasping pattern and motor strategy remained essentially unchanged.

The final child was affected by monodactyly with a hypoplastic thumb and agenesis of the remaining fingers. Surgical planning involved a first-stage free phalanx graft to the thumb, followed by a second-stage double toe transfer. The transferred toes were positioned on the third and fourth metacarpal bones, respectively. During follow-up, the patient predominantly used a bidigital pinch between the thumb and the digit positioned on the third metacarpal bone, successfully manipulating objects of even sub-millimetric dimensions. However, the second transferred toe, placed on the fourth metacarpal bone, was consistently excluded from grasping activities and progressively overlapped with the adjacent digit as growth occurred. The patient subsequently underwent multiple surgical revisions, including tenolysis procedures at 2 and 7 years postoperatively. During the second revision, a derotational osteotomy of the second transferred toe was also performed. Despite multiple surgical revisions, the ulnarly positioned digit continued to function primarily as a stabilizer and remained largely excluded from grasping. At the latest follow-up it exhibited significant stiffness.

### Group B

The female patient affected by monodactyly and agenesis of other digits was planned for a single toe transfer which was positioned on the fifth metacarpal bone. During follow-up, the patient demonstrated a well-coordinated motor pattern for both large object grasping and precision pinch tasks, successfully performing a thumb-transferred digit grip even for objects smaller than one centimeter. Two years postoperatively, she underwent a web space widening procedure, without the need for tenolysis.

**Figure 2:**
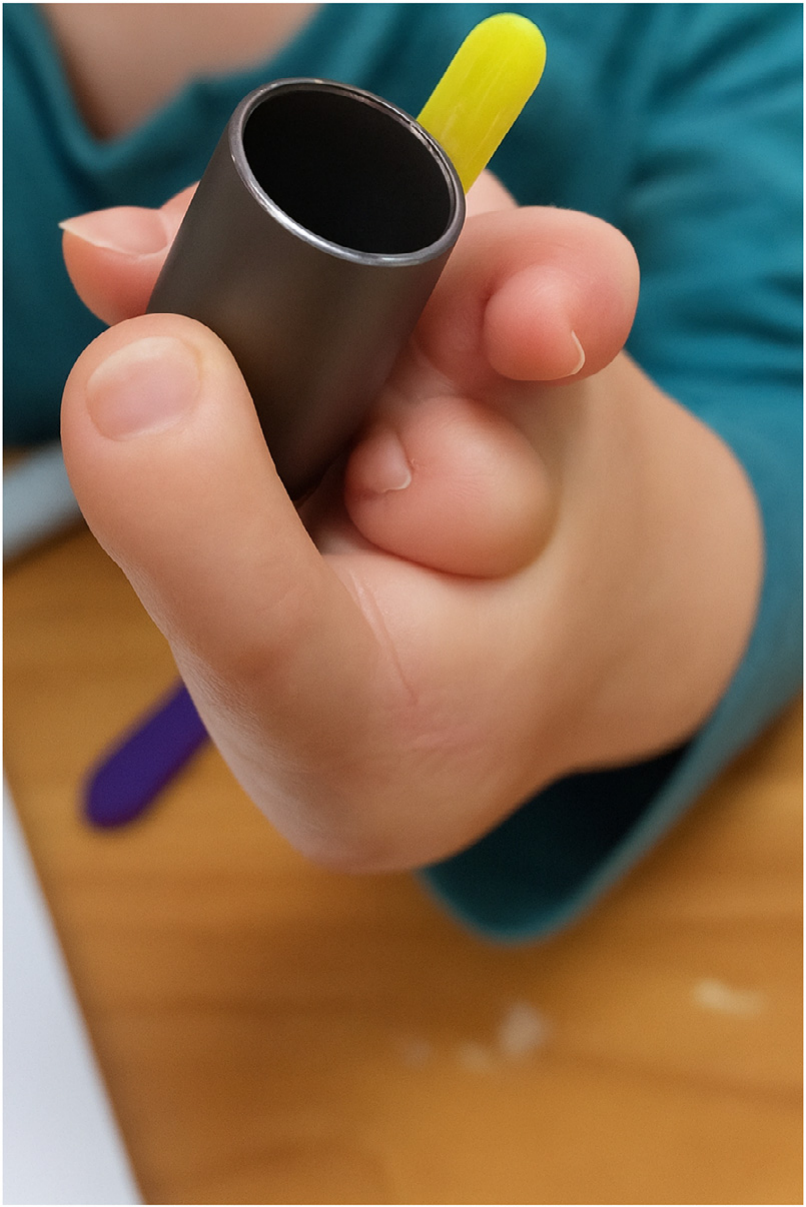
Preferential grasp pattern in patient underwent single toe transfer and free phalangeal transfer. The patient prefers a grasp between the thumb and the transferred digit, using the second hypoplastic finger as a stabilizer when needed.

One of the three remaining children underwent a free phalanx graft to the rudimentary second digit and a single toe transfer to the fifth metacarpal bone. However, radiographic imaging revealed a fusion at the base of the fifth and the fourth metacarpals, which led to functional limitations during follow-up. The transferred digit developed stiffness at the metacarpophalangeal joint, reducing its mobility. Nevertheless, at the most recent follow-up, the patient demonstrated a functional pinch between the thumb and the transferred digit, with the second rudimental digit acting as a stabilizer [Figure 2].

Another child underwent free phalanx graft transfer to the rudimentary second digit and single toe transfer to the third metacarpal bone. No other surgical procedure was necessary. The child used the operated hand for both large objects and fine precision tasks. She predominantly relied on a pinch between thumb and the second rudimental digit, a pattern that was already observed preoperatively; with the progression of physiotherapy this tendency decreased although it remained observable throughout the follow-up period. A double toe transfer was initially planned but the hypothesis was abandoned considering the satisfactory functional results achieved.

The same decision process occurred in the last child, where ultimately a single toe transfer to the third metacarpal bone combined with a free phalanx transfer for the second rudimental digit were performed, since the child tended to use preoperative compensatory movements with an attempt to involve the second digit. In the follow-up, this patient demonstrated alternating use of a thumb-second digit pinch and a thumb-transferred digit pinch providing stabile grasping patterns.

## Discussion

The surgical strategy of restoring functional hand in children affected by symbrachydactyly remains a challenge due to the extreme variability of phenotypes.

Monodactyly is a subtype of symbrachydactyly which severely affects children’s quality of life. The surgical reconstruction typically involves toe-to-hand transfer but the optimal number of digits to be transferred remains a subject of debate. Despite the lack of certain guidelines, some authors have suggested a double toe transfer in order to obtain a tripod pinch. The second transferred digit, in fact, could potentially guarantee a more sophisticated hand function^10^ together with a better grasp stability.^5^ Furthermore, a three-digits hand can have a meaningful psychological impact on children as it offers a hand that more closely resembles a typical one.

Given the limited sample size and the observational nature of our study, we conducted a pilot study aimed at generating preliminary evidence and guiding future research on functional integration following toe transfer in children affected by monodactyly.

Our analysis raises important questions about the actual functional benefit of the second transferred toe. In our clinical experience, in all three patients who underwent double toe transfer a consistent pattern was observed. In each case only one of the transferred digits was fully integrated into fine grasping tasks, while the second digit, whether centrally or ulnarly positioned, was functionally excluded and primarily served as a passive stabilizer. Despite multiple secondary surgical procedures aimed at improving mobility, the excluded digit consistently demonstrated reduced involvement in precision movements and over time developed significant stiffness. In contrast, the integrated digit enabled effective bidigital grasp function in combination with the thumb, especially for large objects. The girl who presented with a rudimentary hypoplastic index which underwent a free phalanx graft, more commonly favored a thumb-index pinch for small objects over the thumb-transferred toe pinch, a pattern already observed preoperatively. This could suggest that residual native anatomy, even if limited, may possess an intrinsic advantage in terms of functional integration. An additional considerable finding is the hierarchy reported by this child when asked to rank her fingers in order of preference.

The central digit was ranked as the least preferred and this may reflect both its limited functional role and a lack of perceived utility or acceptance by the child. Therefore, according to our analysis it seems that the focus should be on preserving and optimizing native structure whether is possible, rather than attempting to reconstruct a new one. On the contrary, when grasping larger objects, all children preferred using the thumb in combination to one of the digits transferred, demonstrating that these patients are able to differentiate between type of grasps and selectively engage specific digits depending on the tasks.

Another important observation is that in a patient who received a single toe transfer, the transferred digit was successfully integrated as the primary functional element for both precision and power grasping, with the rudimentary hypoplastic second digit serving as a stabilizer. Consequently, our findings suggest that hand function does not depend only on the number of the digits but rather by the strategic placement of the transferred digits as well as on functional integration and learned motor strategies. Over reconstruction may, in certain cases, paradoxically limit functional outcomes since a stiff non-functional digit may sometimes represent a greater obstacle than its absence.

Therefore, while double toe transfer can improve grasp and stability, as supported by existing literature, such surgical procedure should be carefully evaluated on patients-specific basis, considering residual anatomy, spontaneous grasp strategies and the potential impact on overall hand function. In addition, we observed from this analysis how preoperative anatomical variants such as metacarpal fusion, may significantly affect the functional outcome of the transferred digits. This underscores the need for detailed skeletal assessment and individualized planning taking into account the expected postoperative range of motion. A patient-centered approach may help prevent the potential exclusion of the transferred digit from the grasp.

## Conclusion

Our findings raise important considerations regarding functional integration of transferred digits in children affected by monodactyly. While double toe transfer can offer enhanced functional outcomes in select cases, such surgical procedure is associated with extended operative time and higher risk of donor site morbidity as compared with single toe transfer. In our study, in double toe transfer, one of the two transferred toes consistently has appeared to be excluded from active grasp, with a limited spontaneous use of the digit by the child. These results suggest that functional integration of the transferred toe into the motor schema of the child is fundamental for the success of the procedure. A vital and sensate digit does not appear to be sufficient to ensure its involvement in daily grasping tasks.

In our series the presence of a well-structured thumb and a single toe transfer appeared to be sometimes sufficient to establish a stable grasp in specific patient.

Consequently, this work supports the need for a more selective, individualized approach rather than a routine double transfer strategy. Future studies should aim to develop a patient-centered approach to establish predictive markers that could guide surgical planning, optimizing surgical timing while also minimizing donor site morbidity if unnecessary. The potential functional benefits of adding an additional digit should always be balanced against the risk of increased donor site morbidity. We believe that the predictive model should be potentially based on pre-operative assessment of spontaneous grasp strategies which could suggest if and how additional digits will be functionally integrated. Observing how children utilize their residual digits prior to surgery may suggest if these patients will exclude the second transferred toe, since a rudimentary but still functional index finger could be preferred. Early intervention may favor a more efficient functional integration together with a structured rehabilitation program.

This study has several limitations. First, it is a retrospective observational analysis which, due to the rarity of monodactyly, has a limited sample size. This may reduce the generalizability of the findings. Second, functional evaluations were performed in absence of a standardized evaluation scale. However, although functional assessment scales such as the Pediatric Outcomes Data Collection Instrument can provide information on global upper limb function, they are not specific for evaluating the integration of the transferred toes into grasp. Consequently, such scales cannot clarify whether global hand function actually involves the active use of the transferred digits or rather results from compensatory strategy. Although the limited sample size does not allow definitive conclusions to be drawn, this pilot study may provide valuable preliminary insights into functional relevance of single versus double toe transfer in patients with monodactyly.

## Funding sources

This research did not receive any specific grant.

## Ethical approval

Not required.

## Declaration of AI and AI-assisted technologies in the writing process

During the preparation of this work the authors used ChatGPT (OpenAI) in order to improve the clarity and language of the English text. After using this tool/service, the authors reviewed and edited the content as needed and take full responsibility for the content of the publication.

## Declaration of competing interest

Authors have no conflict of interest to declare.
